# Getting everyone to agree on gene signatures for murine macrophage polarization *in vitro*

**DOI:** 10.1371/journal.pone.0297872

**Published:** 2024-02-08

**Authors:** Giorgia Colombo, Emanuela Pessolano, Maria Talmon, Armando A. Genazzani, Paolo Kunderfranco

**Affiliations:** 1 Department of Pharmaceutical Sciences, Università del Piemonte Orientale, Novara, Italy; 2 Bioinformatics Unit, IRCCS Humanitas Research Hospital, Rozzano, Milan, Italy; Umass Chan Medical School, UNITED STATES

## Abstract

Macrophages, key players in the innate immune system, showcase remarkable adaptability. Derived from monocytes, these phagocytic cells excel in engulfing and digesting pathogens and foreign substances as well as contributing to antigen presentation, initiating and regulating adaptive immunity. Macrophages are highly plastic, and the microenvironment can shaper their phenotype leading to numerous distinct polarized subsets, exemplified by the two ends of the spectrum: M1 (classical activation, inflammatory) and M2 (alternative activation, anti-inflammatory). RNA sequencing (RNA-Seq) has revolutionized molecular biology, offering a comprehensive view of transcriptomes. Unlike microarrays, RNA-Seq detects known and novel transcripts, alternative splicing, and rare transcripts, providing a deeper understanding of genome complexity. Despite the decreasing costs of RNA-Seq, data consolidation remains limited, hindering noise reduction and the identification of authentic signatures. Macrophages polarization is routinely ascertained by qPCR to evaluate those genes known to be characteristic of M1 or M2 skewing. Yet, the choice of these genes is literature- and experience-based, lacking therefore a systematic approach. This manuscript builds on the significant increase in deposited RNA-Seq datasets to determine an unbiased and robust murine M1 and M2 polarization profile. We now provide a consolidated list of global M1 differentially expressed genes (*i*.*e*. robustly modulated by IFN-γ, LPS, and LPS+ IFN-γ) as well as consolidated lists of genes modulated by each stimulus (IFN-γ, LPS, LPS+ IFN-γ, and IL-4).

## 1. Introduction

Macrophages are pivotal components of the innate immune system, known for their remarkable versatility in orchestrating immune responses [1]. Derived from monocytes, these phagocytic cells exhibit an exceptional ability to engulf and digest pathogens, cellular debris, and foreign substances [2]. Their functions extend beyond mere phagocytosis, as they play a crucial role in antigen presentation, contributing to the initiation and regulation of adaptive immunity [3].

Macrophages are highly plastic [4, 5] and the microenvironment can shape their phenotype: the two ends of the spectra are classical activated macrophages (M1-polarizated, type I subset; inflammatory) and alternative activated macrophages (M2-polarizated, type II subset; anti-inflammatory), both characterized by a specific and fine molecular and transcriptomic pattern [6]. Classical activated macrophages can be polarized by different inflammatory stimuli such as Th1-related cytokines including interferon-γ (IFN-γ) and tumour necrosis factor-α (TNF-α), alone or in combination with microbial products such as lipopolysaccharide (LPS) or lipoteichoic acid. This leads to the production of pro-inflammatory cytokines, oxidative species and proteases. Though essential in host defence, against pathogens and tumoral cells, M1 macrophages also contribute to several autoimmune diseases, determining tissue damage and organ loss of function [6, 7]. On the contrary, M2 macrophages can be polarized by IL-4 and IL-13, which are classically produced by Th2-polarized T cells [7, 8]. M2 macrophages participate, among other functions, in wound healing, tissue repair, as well as fibrosis, airway hypersensitivity, and also helminthic infections [9]. While of importance for reductionist research, M1 and M2 are two of the many polarized subsets that may exist (according to the stimulus, the microenvironment, etc) [10, 11], and, to this end, M1/M2 mixed phenotypes have also been described [12].

M1 and M2 macrophages exhibit distinct gene expression signatures, reflecting their diverse functions and roles. M1 macrophages, often associated with pro-inflammatory responses, are characterized by the upregulation of genes such as IL-1β, IL-6, TNF-α, and inducible nitric oxide synthase (iNOS). These molecules play essential roles in host defence against infections and the promotion of tissue damage during inflammatory conditions [9, 13]. In contrast, M2 macrophages, considered anti-inflammatory and tissue repair-promoting, display an expression profile marked by genes like Arginase-1 (Arg1), IL-10, and the mannose receptor (CD206). These genes are associated with immunomodulation, extracellular matrix remodelling, and wound healing [7, 9, 13, 14].

The advent of RNA sequencing (RNA-Seq) represents a revolutionary breakthrough in molecular biology and genomics. This technology has ushered in a new era of high-throughput transcriptome analysis, providing researchers with an unprecedented level of insight into gene expression patterns and regulation [15]. Unlike microarray technology, RNA-Seq offers a more comprehensive and quantitative view of the transcriptome. It enables the detection of both known and novel transcripts, alternative splicing events, and rare transcripts, facilitating a deeper understanding of the functional complexity of the genome [15]. Moreover, RNA-Seq is highly sensitive and can quantify gene expression across a wide dynamic range [16].

The rapid decrease in costs associated with RNA-seq has significantly increased the number of datasets deposited in public databases and this is even more true for macrophages. Yet, to some surprise, there is no attempt to consolidate the information coming from the different murine or human datasets. This would have the advantage of reducing the intrinsic noise and allowing for authentic signatures, not driven by the scientific context and question.

M1 and M2 gene expression signatures are traditionally defined from historical findings. While this approach undoubtably is based on biologically- relevant genes it lacks a systematic approach to define the most robust possible signature. In 2015, Jablonski *et al*. applied a systematic approach to better define murine M1 and M2 polarization signatures [17] from a single microarray dataset. In the present manuscript, we took a step further and combined all the published murine RNA-Seq databases deposited so far to portray a robust polarization profile.

## 2. Materials and methods

### 2.1 Datasets and exclusion criteria

The NIH Gene Expression Omnibus (GEO) database was thoroughly queried to pinpoint datasets encompassing whole-genome transcriptome information of macrophages. We have used the following string: *(("macrophages"[MeSH Terms] OR macrophages[All Fields]) AND “**treatment*”*[All Fields]) AND "Mus musculus"[porgn] AND "Expression profiling by high throughput sequencing"[Filter] (*“*treatment**”* stands for LPS, IFNγ, ect).

We excluded those studies whose data were not available on Sequence Read Archive (SRA).

### 2.2. RRA discovery study–Identification of robust differentially expressed genes by RNA-seq data RRA method

To integrate results from multiple datasets, we employed the robust rank aggregation (RRA) method, a well-established tool for analysing data from diverse arrays [18]. Initially, we conducted an RRA discovery study using data extracted from all RNA-Seq datasets available. For each array dataset, we downloaded both the raw fastq files and the associated annotation document from the NCBI Sequence Read Archive (SRA) repository.

For each dataset, raw fastq files were processed through the nf-core/RNA-Seq pipeline version 3.6 [19] and mapped using star_rsem aligner against the mouse reference genome GRCm38. FeatureCounts module from the subread package was used for counting reads associated to genomic features. To compute differential expression analysis based on the negative binomial distribution independently for each dataset, we applied ‘DESeq2’ Bioconductor R package [20]; only genes with an adjusted *p*-value ≤0.05 and a fold change greater than |1.5| were selected for further analysis.

For each signature, the generated lists of differentially up-regulated and down-regulated genes in each dataset were then aggregated using the ‘Robust Rank Aggregation’ Bioconductor R package. Only genes with a *p*-value≤0.05 (RRA score) and whose fold-change was conserved in each independent dataset were retained in the final signature. Complete lists of signatures are listed in S1 and S2 Files.

### 2.3. Ingenuity pathway analysis and IPA comparison analysis

For each signature, functional enrichment of differentially expressed genes was performed using IPA (QIAGEN). Pathways belonging to the Canonical Pathway Analysis section were considered for the analysis (-log_10_ *p*-value ≥1.3).

## 3. Results

### 3.1 Unravelling the expression signatures of M1 and M2 macrophages

**Fig 1:**
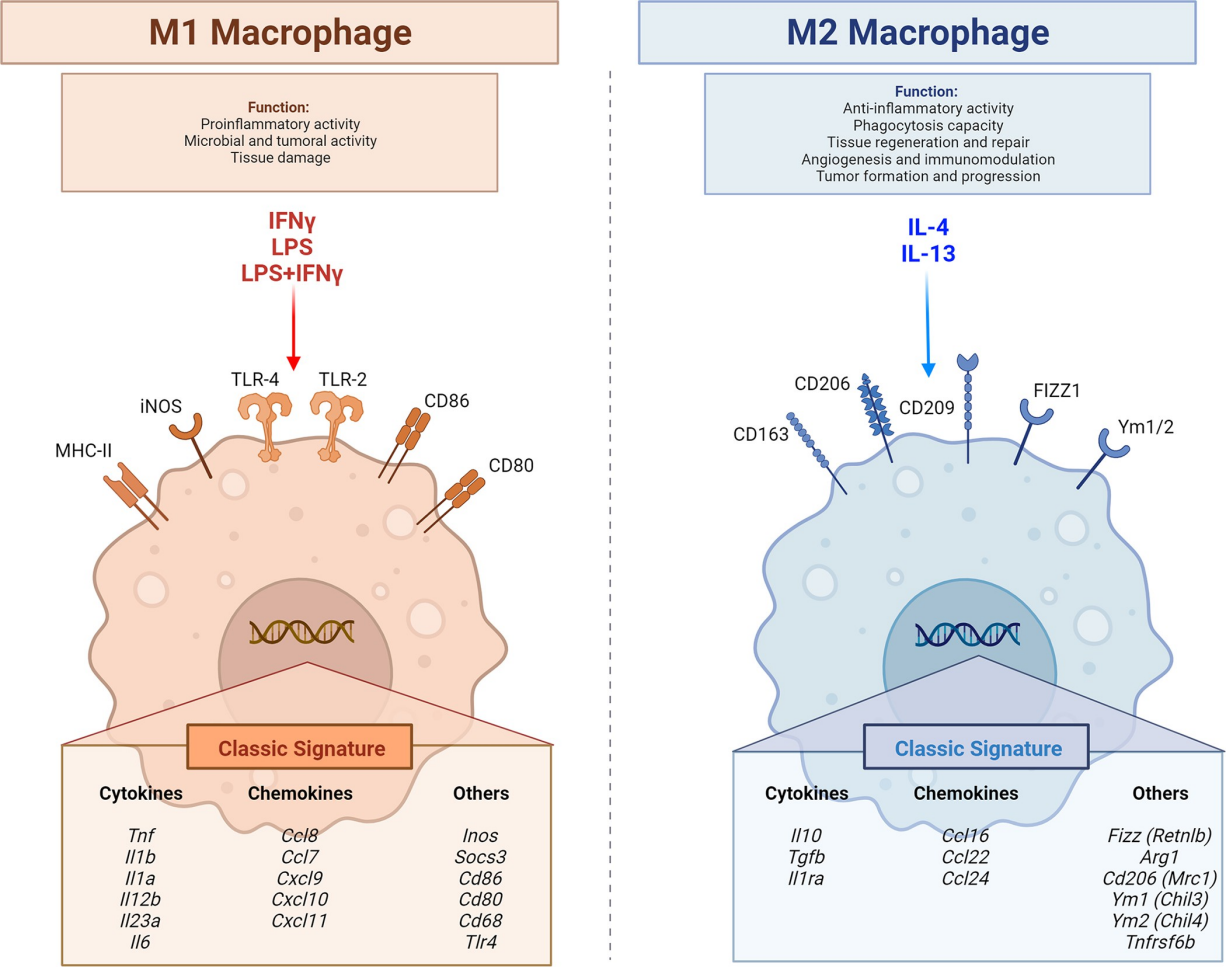
Cartoon depicting M1 and M2 characteristics, including the classical genes usually used to determine polarization.

M1 and M2 polarizations are traditionally investigated by the up-regulation of genes which have been historically associated with these phenotypes (Fig 1).

In the present manuscript, as we wanted an unbiased approach, we first screened to find eligible RNA-Seq datasets for the most commonly used polarizing stimuli: IFNγ, LPS, IFNγ+LPS, IL-4 and IL-13. For IFNγ 5 datasets were eligible, for LPS 12 datasets were eligible, for IFNγ+LPS 13 were eligible, for IL-4 19 were eligible (Table 1). For IL-13 only 2 datasets were found and this was deemed insufficient to perform further meaningful analysis. While other stimuli are known to induce an M1 or M2 polarization [21–25], we chose those described above as they would have been more likely to yield enough datasets to meta-analyse. The analysis was built around rank-robust analysis (RRA) and fold-change. Data in the main text represent the top 20 up-regulated genes for each analysis, while the full analysis (all genes up- and down-regulated) is presented in the supplementary files (S1 File and Supplementary Figs 1–4 in S5 File).

**Table 1:** Publicly available murine datasets used in the present study. BMDMs: bone marrow-derived macrophages. PECs: peritoneal exudate cells.

*GSE ID*	*CELL SOURCE*	*TREATMENT*	*TIMING*	*TYPE OF ANALYSIS*	*PLATFORM*
**GSE120807**	BMDMs	LPS, IFN-γ, LPS+IFN-γ	4–8 h	RNA-Seq	Illumina HiSeq 2500
**GSE189104**	PECs	IFN-γ	4 h	RNA-Seq	Illumina NextSeq 500
**GSE120808**	BMDMs	LPS+IFN-γ, IFN-γ, LPS	4–8 h	RNA-Seq	Illumina HiSeq 2500
**GSE116904**	BMDMs	IFN-γ, IL-4	8/24h	RNA-Seq	Illumina HiSeq 2500
**GSE145243**	BMDMs	IFN-γ, IL-4	7/24 h	RNA-Seq	Illumina NovaSeq 6000
**GSE84517**	BMDMs	IFN-γ, IL-4	4 h	RNA-Seq	Illumina HiSeq 2000
**GSE184551**	BMDMs	LPS	2 h	RNA-Seq	Illumina NextSeq 500
**GSE82087**	Peripheral macrophages	LPS	4 h	RNA-Seq	Illumina HiSeq 2000
**GSE103958**	BMDMs and RAW264.7	LPS, LPS+IFN-γ IL-13+IL-4	4/12 h	RNA-Seq	Illumina HiSeq 2500
**GSE142088**	RAW264.7	LPS	12 h	RNA-Seq	Illumina NovaSeq 6000
**GSE56123**	BMDMs	IFN-γ, LPS	1-2-4 h	RNA-Seq	Illumina HiSeq 2000
**GSE196680**	PECs	LPS	6 h	RNA-Seq	Illumina NextSeq 500
**GSE160246**	BMDMs	LPS	1-3-6-9 h	RNA-Seq	Illumina HiSeq 2500
**GSE152241**	BMDMs	LPS	4 h	RNA-Seq	HiSeq X Ten
**GSE113594**	BMDMs	LPS, IL-4	4/24 h	RNA-Seq	Illumina HiSeq 2000
**GSE123180**	BMDMs	LPS, IL-4	4/24 h	RNA-Seq	Illumina HiSeq 2000
**GSE123596**	BMDMs	LPS+IFN-γ, LPS, IL-4	1/18 h	RNA-Seq	Illumina HiSeq 3000
**GSE152700**	BMDMs	LPS + IFNγ	1 h	RNA-Seq	Illumina NovaSeq 6000
**GSE188917**	BMDMs	LPS + IFNγ	12 h	RNA-Seq	Illumina NovaSeq 6000
**GSE155566**	RAW264.7	LPS+IFN-γ	24 h	RNA-Seq	HiSeq X Ten
**GSE171270**	BMDMs	LPS+IFN-γ	24 h	RNA-Seq	Illumina NextSeq 500
**GSE158094**	BMDMs	LPS+IFN-γ IL-4	6/24 h	RNA-Seq	Illumina NextSeq 500
**GSE145523**	BMDMs	LPS+IFN-γ	18 h	RNA-Seq	Illumina HiSeq 3000
**GSE112595**	PECs	LPS+IFN-γ	12 h	RNA-Seq	Illumina HiSeq 2500
**GSE103958**	BMDMs and RAW264.7	LPS, LPS+IFN-γ IL-13+IL-4	4/18 h	RNA-Seq	Illumina HiSeq 2500
**GSE172119**	BMDMs	LPS+IFN-γ	18 h	RNA-Seq	Illumina HiSeq 2500
**GSE123596**	BMDMs	LPS+IFN-γ, LPS, IL-4	1/18 h	RNA-Seq	Illumina HiSeq 3000
**GSE145720**	BMDMs	LPS+IFN-γ, IL-4	12 h	RNA-Seq	Illumina NovaSeq 6000
**GSE148948**	BMDMs	LPS+IFN-γ IL-4	24 h	RNA-Seq	Illumina NextSeq 500
**GSE195438**	BMDMs	IL-4	24 h	RNA-Seq	Illumina HiSeq 4000
**GSE168542**	BMDMs	IL-4	16 h	RNA-Seq	Illumina HiSeq 2500
**GSE58318**	BMDMs	IL-4	48 h	RNA-Seq	Illumina HiSeq 2000
**GSE158510**	BMDMs	IL-4, IL-13	24 h	RNA-Seq	Illumina HiSeq 2500
**GSE151015**	BMDMs	IL-4	24 h	RNA-Seq	Illumina NextSeq 500
**GSE151213**	BMDMs	IL-4	24 h	RNA-Seq	Illumina HiSeq 2500
**GSE58283**	BMDMs	IL-4	72 h	RNA-Seq	Illumina HiSeq 2500

**Fig 2:**
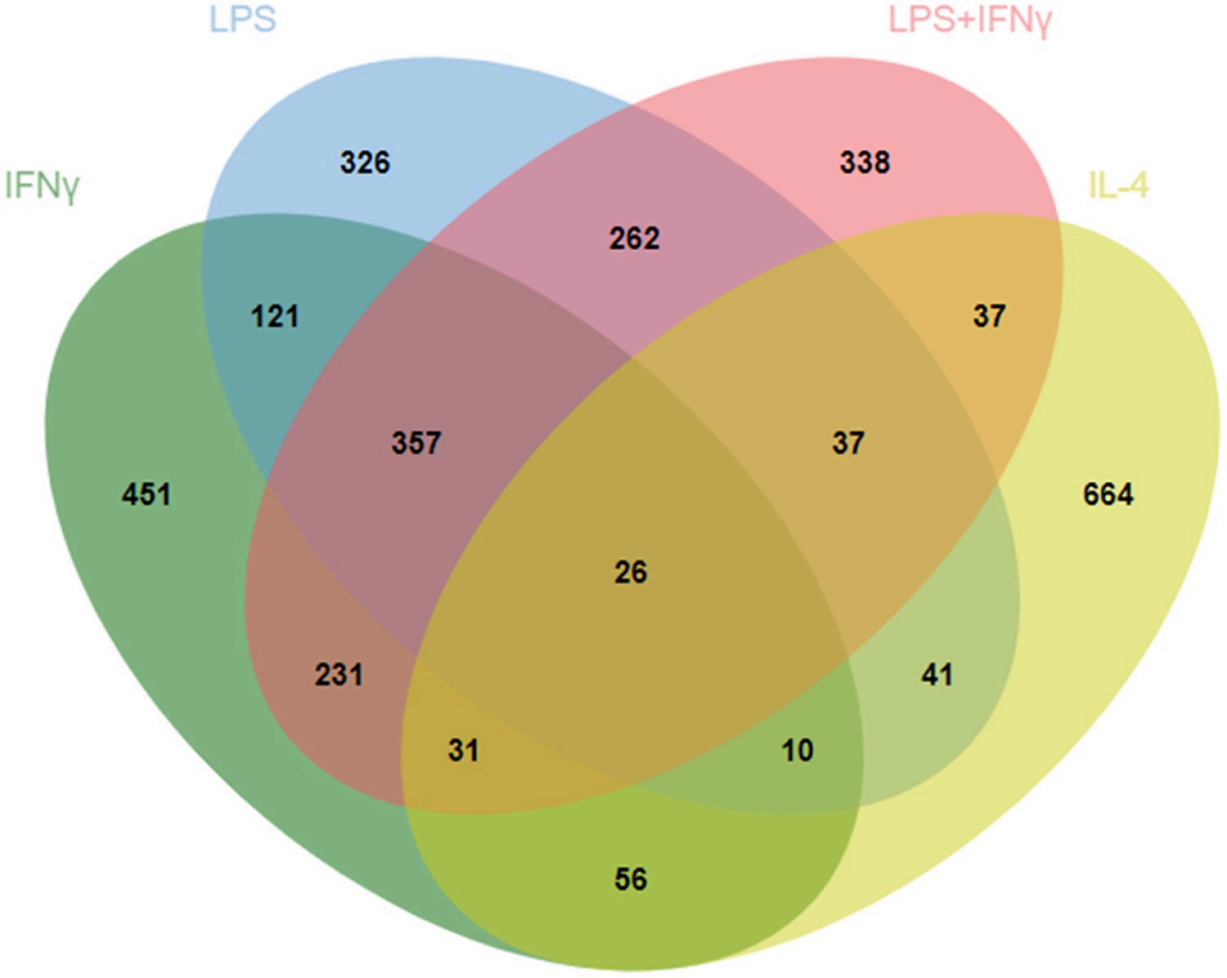
Interpolation of the RRA analyses. Four-set Venn diagram analysis of the statistically significant (p<0.05) genes emerging from the individual RRA analyses performed with Jvenn [29].

The RRA analysis returned 1283 significant (p<0.05) genes for IFNγ, 1180 genes for LPS, 1319 for LPS+IFNγ and 902 genes for IL-4. As shown in the Venn diagram in Fig 2, a very high proportion of significant genes were unique to the selected stimuli but, as expected, there were a high number of genes shared by the three M1-inducing stimuli (LPS, IFNγ, LPS+IFNγ). As an example, when evaluating the genes up-regulated by IFNγ, 451 genes were solely found significantly and robustly modulated in the IFNγ dataset, in contrast to 121 which were shared with the LPS dataset and 357 which were shared with the LPS+IFNγ dataset. To improve the robustness of the M1 signature over the historical one, we arbitrarily selected those genes that showed a p<10^−7^ in each of the three datasets and that were not present in the RRA list for IL-4. The 28 genes resulting from this analysis together with the respective fold-changes are listed in Table 2 and can be viewed as an M1-restricted and consolidated list. We performed a similar analysis using the fold-change as a parameter, setting the cut-off to log2 fold-change > 5. Eleven genes were in common for all three M1 stimuli (Table 3). Few genes, as expected, emerged as shared between the M2-polarizing agent IL-4 and the other three stimuli, both when analyzing RRA or fold-change.

**Table 2:** Genes with a RRA p<10^−7^ in the LPS, IFNγ, LPS+IFNγ datasets not present in the IL-4 dataset.

	*avgFC (IFNγ)*	*avgFC (LPS)*	*avgFC (LPS+IFNγ)*
***Irf1***	4.7 ± 0.3	2.2 ± 0.6	3.7 ± 0.7
***Gbp5***	7.7 ± 0.4	3.7 ± 0.9	8.4 ± 1.1
***Batf2***	6.2 ± 0.3	3.3 ± 0.7	5.1 ± 0.9
***Gbp2***	7.6 ± 0.8	4.1 ± 0.8	7.4 ± 1.0
***Irgm1***	4.0 ± 0.4	1.9 ± 0.5	3.7 ± 0.6
***Igtp***	4.8 ± 0.5	6.0 ± 0.9	4.5 ± 0.6
***Gbp3***	5.6 ± 0.5	2.5 ± 0.8	5.9 ± 0.9
***Nampt***	2.8 ± 0.2	1.6 ± 0.3	2.6 ± 0.4
***Serpina3g***	10.8 ± 1.1	4.3 ± 0.9	9.5 ±1.2
***Gbp7***	5.0 ± 0.3	3.3 ± 0.6	5.2 ± 0.6
***Cxcl10***	6.9 ± 0.8	4.4 ± 0.1	8.5 ± 1.2
***Nod1***	3.3 ± 0.2	2.7 ± 0.4	3.2 ± 0.7
***Gbp6***	7.9 ± 1.3	4.5 ± 0.9	8.2 ± 0.9
***Tap1***	3.2 ± 0.3	2.3 ± 0.3	3.4 ± 0.5
***Parp9***	2.2 ± 0.2	1.8 ± 0.4	2.4 ± 0.4
***Gbp9***	4.7 ± 0.4	2.8 ± 0.3	3.6 ± 0.6
***Casp4***	2.8 ± 0.2	2.4 ± 0.5	3.1 ± 0.4
***Gbp4***	10.0 ± 1.3	4.4 ± 0.1	10.2 ± 1.0
***Irgm2***	3.7 ± 0.4	2.2 ± 0.4	3.6 ± 0.4
***Pla2g4a***	2.0 ± 0.2	1.6 ± 0.4	2.4 ± 0.4
***Nlrc5***	3.1 ± 0.3	2.3 ± 0.3	3.0 ± 0.5
***Sp140***	2.3 ± 0.3	1.5 ± 0.7	2.5 ± 0.4
***Peli1***	1.9 ± 0.3	2.1 ± 0.7	2.4 ± 0.4
***Serpina3f***	12.0 ± 1.3	4.1 ± 0.1	9.2 ±1.6
***Slco3a1***	5.1 ± 0.6	3.2 ± 0.5	5.8 ± 0.6
***Casp1***	1.6 ± 0.1	1.5 ± 0.3	2.0 ± 0.2
***Mlkl***	2.3 ± 0.3	2.3 ± 0.3	3.1 ± 0.4
***Il27***	5.0 ± 0.6	3.7 ± 0.2	6.7 ± 1.0

**Table 3:** Genes with a log2 fold-change > 5 in the LPS, IFNγ, LPS+IFNγ datasets not present in the IL-4 dataset.

	*RRA (IFNγ)*	*RRA (LPS)*	*RRA (LPS+IFNγ)*
***Gbp5***	1.01E-12	1.18E-19	2.24E-12
***Ptgs2***	2.36E-06	2.79E-14	2.51E-12
***Gbp2***	4.29E-12	6.62E-15	3.26E-17
***Cxcl10***	1.03E-09	5.43E-16	1.40E-09
***Socs1***	4.83E-08	5.69E-15	2.32E-12
***Calhm6***	1.79E-10	1.60E-13	5.33E-13
***Ifi205***	2.40E-07	2.41E-17	1.72E-08
***Gbp3***	8.25E-11	3.96E-17	2.32E-15
***Cd69***	1.80E-07	1.75E-15	6.47E-09
***Acod1***	2.04E-07	5.71E-18	6.18E-17

**Fig 3:**
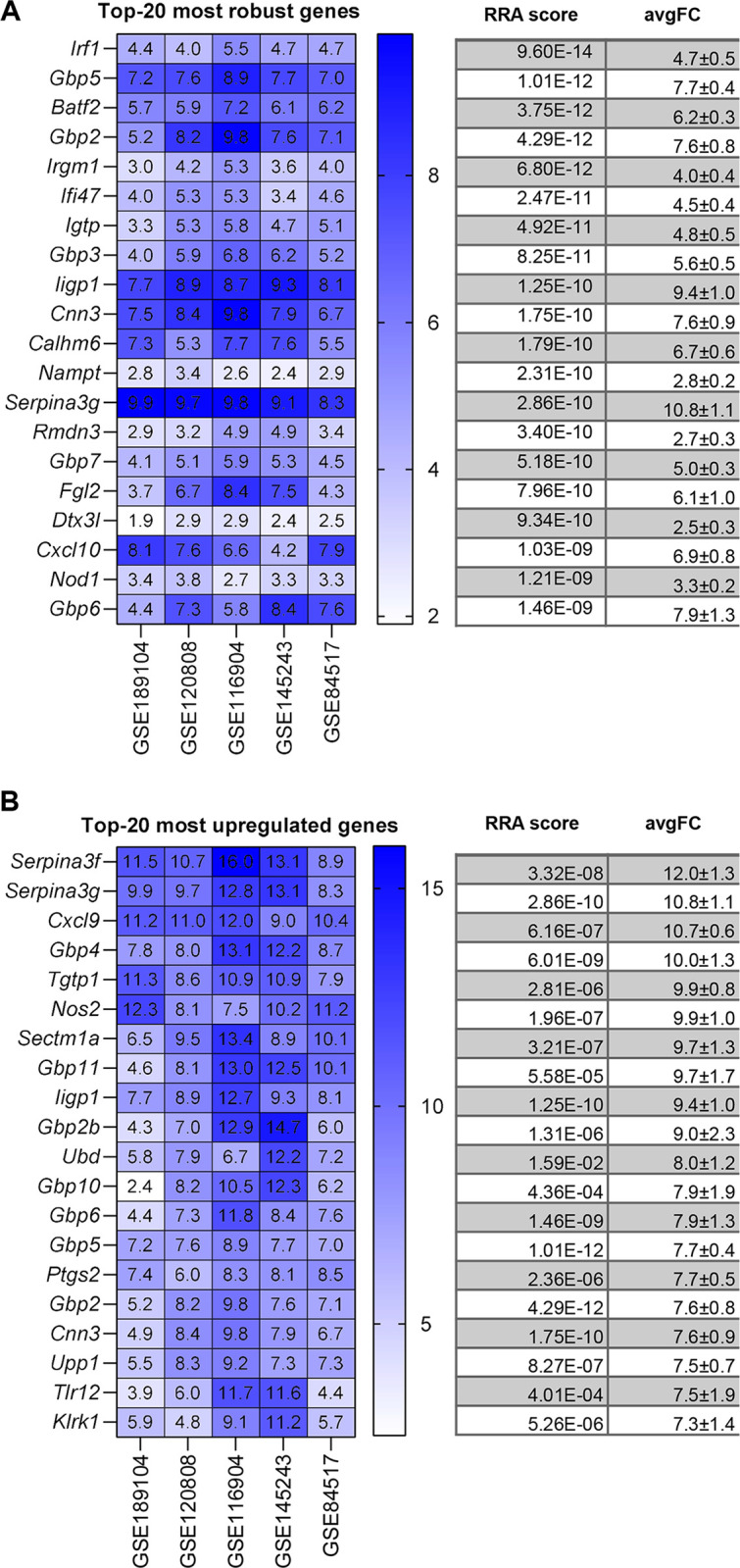
IFNγ up-regulated DEGs identified by RRA and fold-change analysis. (**A**) On the left, the heatmap of the five datasets showing the top 20 most robust genes upregulated is depicted. Value in the boxes represents fold-change and shade of blue represents RRA score. On the right, the corresponding average RRA score and fold-change. (**B**) On the left, the heatmap of the five datasets showing the top 20 most upregulated genes which also present a significant RRA score. Value in the boxes represents fold-change and shade of blue represents RRA score. On the right, the corresponding average RRA score and fold-change.

**Fig 4:**
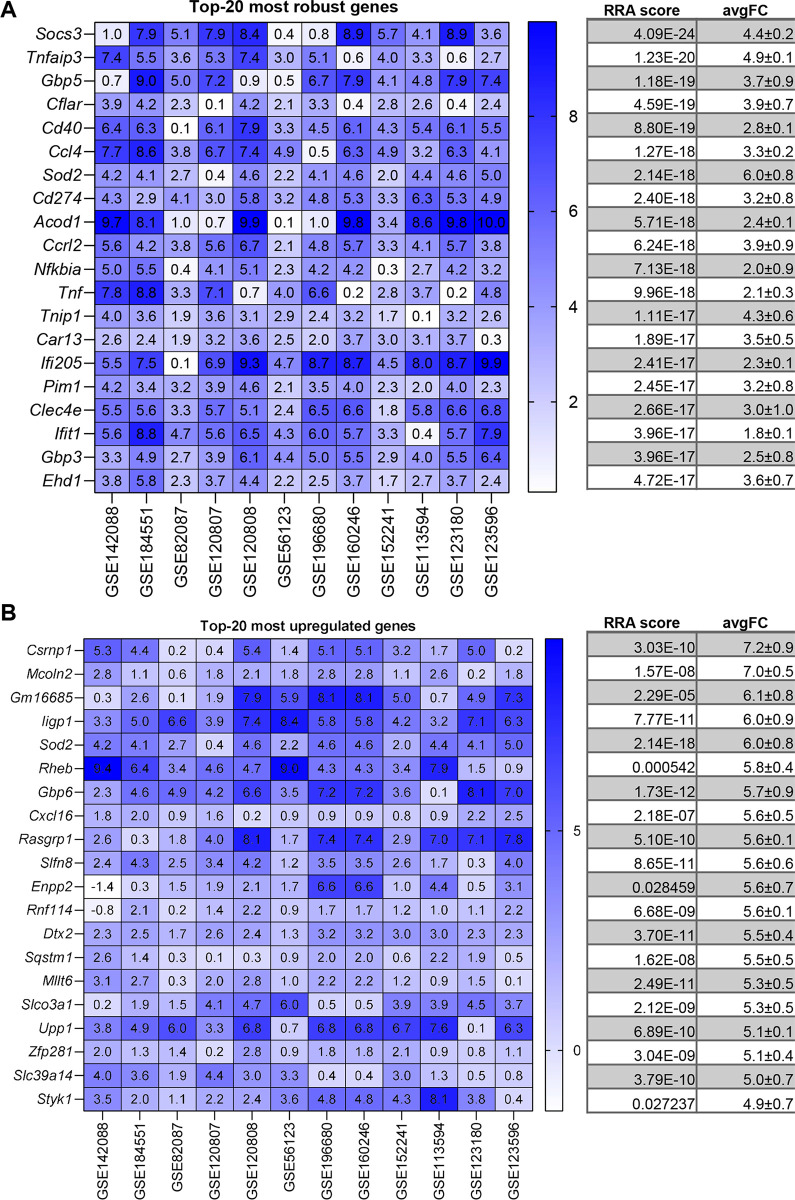
LPS up-regulated DEGs identified by RRA and fold-change analysis. (**A**) On the left, the heatmap of the twelve datasets showing the top 20 most robust genes upregulated is depicted. Value in the boxes represents fold-change and shade of blue represents RRA score. On the right, the corresponding average RRA score and fold-change. (**B**) On the left, the heatmap of the twelve datasets showing the top 20 most upregulated genes which also present a significant RRA score. Value in the boxes represents fold-change and shade of blue represents RRA score. On the right, the corresponding average RRA score and fold-change.

**Fig 5:**
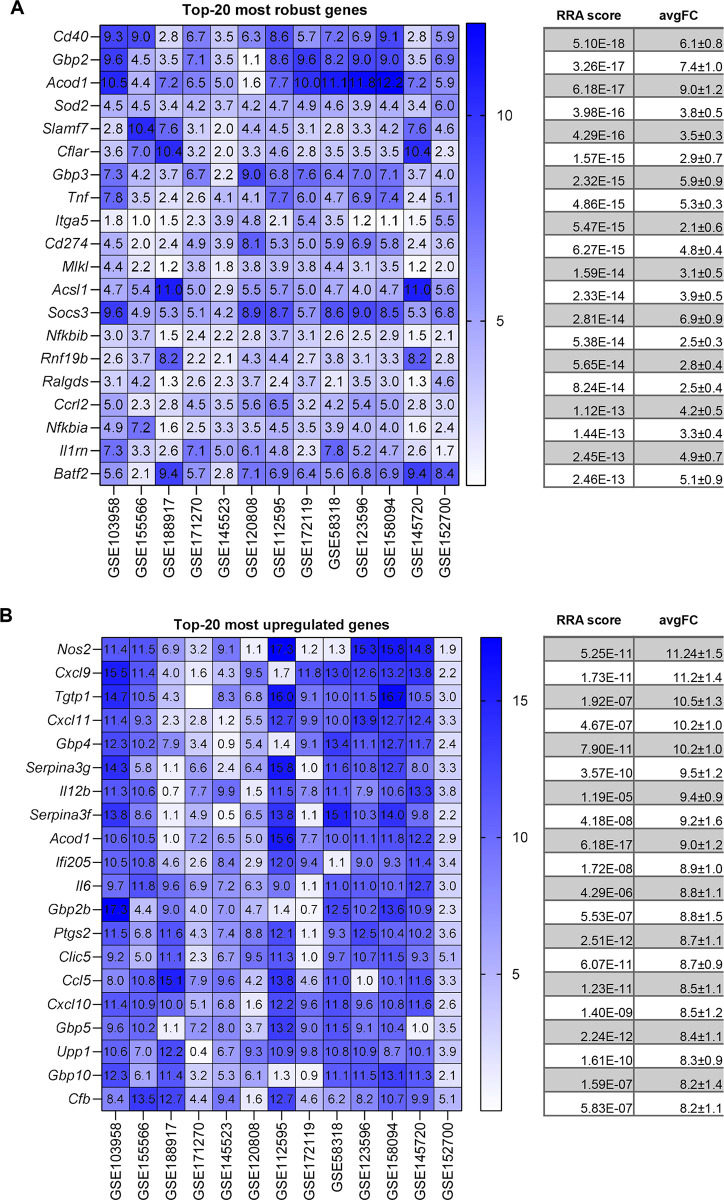
IFNγ+LPS up-regulated DEGs identified by RRA and fold-change analysis. (**A**) On the left, the heatmap of the thirteen datasets showing the top 20 most robust genes upregulated is depicted. Value in the boxes represents fold-change and shade of blue represents RRA score. On the right, the corresponding average RRA score and fold-change. (**B**) On the left, the heatmap of the thirteen datasets showing the top 20 most upregulated genes which also present a significant RRA score. Value in the boxes represents fold-change and shade of blue represents RRA score. On the right, the corresponding average RRA score and fold-change.

**Fig 6:**
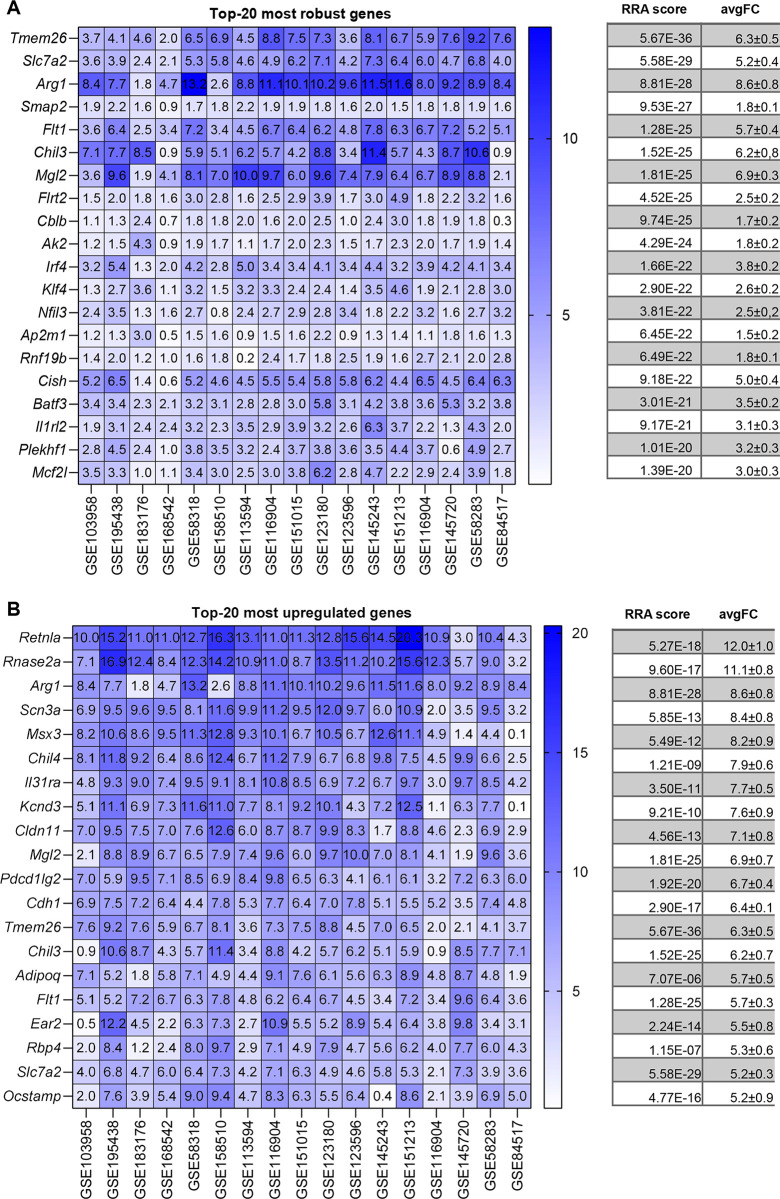
IL-4 up-regulated DEGs identified by RRA and fold-change analysis. (**A**) On the left, the heatmap of the seventeen datasets showing the top 20 most robust genes upregulated is depicted. Value in the boxes represents fold-change and shade of blue represents RRA score. On the right, the corresponding average RRA score and fold-change. (**B**) On the left, the heatmap of the seventeen datasets showing the top 20 most upregulated genes which also present a significant RRA score. Value in the boxes represents fold-change and shade of blue represents RRA score. On the right, the corresponding average RRA score and fold-change.

We next proceeded to analyse each stimulus separately. Figs 3A, 4A, 5A and 6A show the most robust gene changes derived from the overall RRA discovery study (S2 File). It can be noticed that when comparing the classical list with the top 20 robust genes, some are shared, while the majority is distinct.

Briefly, for IFNγ 3 genes are present on both lists (*Irf1*, *Cxcl10*, *Nod1)*, for LPS there are 3 genes (*Socs3*, *Ccl4*, *Tnf)*, for LPS+IFNγ there are 3 genes (*Cd40*, *Tnf*, *Socs3*) while for IL-4 there is a single gene (*Arg1*, *Chil3*).

A similar situation applied for the highest fold-change genes (Figs 2B, 3B, 4B and 5B). Briefly, comparing the “historical” gene signature to the top 20 unbiased aggregated list, 2 genes were in common for IFNγ (*Cxcl9*, *Nos2*), no gene for LPS, 6 genes for LPS+IFNγ (*Nos2*, *Cxcl9*, *Cxcl10*, *Cxcl11*, *Il12b*, *Il6*), and 3 genes for IL-4 (*Arg1*, *Chil3* and *Mrc1*).

### 3.2 IPA UP-stream comparison analysis

**Fig 7:**
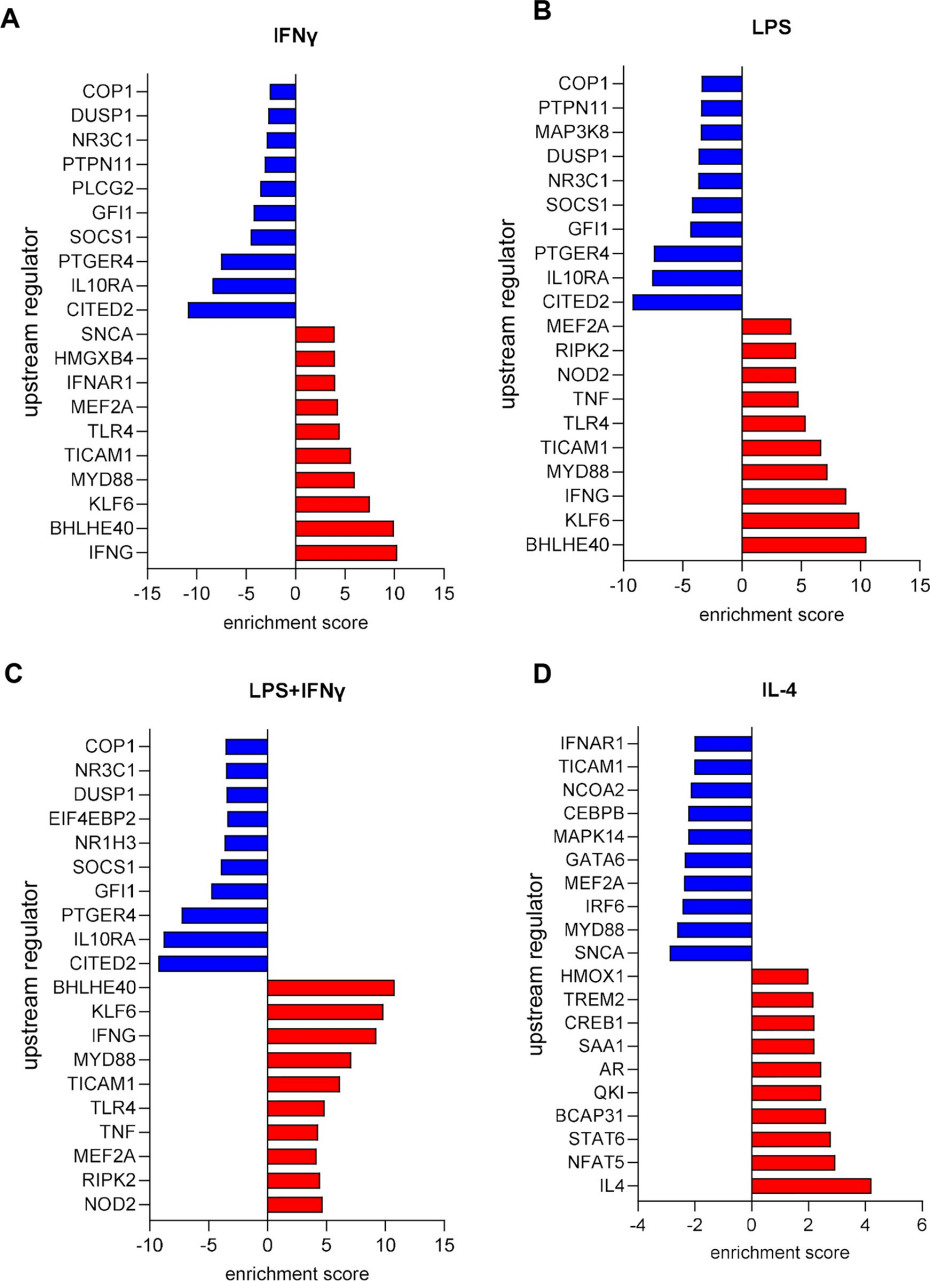
Ingenuity pathway analysis (IPA) of differentially expressed genes in M1- and M2-macrophages. Upstream regulator analysis of differentially expressed genes in (**A**) IFNγ- (**B**) LPS- (**C**) LPS+IFNγ- (**D**) IL-4-treated macrophages.

Last, we compared the pathway and functional enrichment results of the M1- and M2-related treatment and observed significant differences in biological functions related to immune cell functions. IPA upstream regulator analysis provides a powerful tool to predict the deregulated functional activities that are possibly affected by the transcriptome data. Fig 7 describes the top-10 upstream regulators predicted to be activated or inhibited and the significant enrichment score (the complete analysis is present in the S3 File). For IFNγ stimulation (Fig 7A), we observed an activation of regulators (MYD88, IFNG and TLR4) associated to the Macrophage Classical Activation Signalling Pathway (Z score = 3.92 S4 File), as for LPS (Fig 7B). Moreover, LPS also activated regulators (TNF, MYD88, and IFNG) associated to Pathogen Induced Cytokine Storm Signalling Pathway (Z score = 6.04, S4 File). These activated pathways were also observed in the LPS+ IFNγ stimulation (Z score 3.91 and 5.21 respectively), whose regulators were TICAM1, TNF, TLR4 and NOD2. For IL-4, we found a significant enrichment in Macrophage Alternative Activation Signalling Pathway (Z score = 5.02, S4 File), whose regulators were IL-4, TREM2 and STAT6.

## 4. Discussion

RNA-Seq has become a standard experimental procedure to delineate the action of polarizing agents on macrophages. This is evident from the high number of datasets we retrieved from public repositories, when searching for those we reckoned are the most common stimuli used in murine research (IFNγ and/or LPS for M1 and IL-4 and/or IL-13 for M2). Indeed, we were able to find a sufficient number of repeated datasets for three out of these four stimuli (as well as for IFNγ+LPS). Given that it is counter-intuitive to repeatedly perform identical experiments across laboratories without at least attempting to consolidate the information to slimline results and find common trends that reduce experimental noise and chance-findings, we proceeded to meta-analyse the retrieved results. Such effort could yield a smaller, less-time consuming, more robust and cheaper set of genes to investigate polarization as well as reduce animal experiments. It could also yield a set of genes that, when present in RNA-Seq experiments, could be easily associated to a particular stimulus or polarization.

Laboratories working on macrophage polarization, when not performing RNA-Seq, pick selected genes which are known in the literature to be associated with either M1 or M2 polarization. Such genes are chosen because of their important inflammatory function associated with polarization, of their perceived solidity regarding changes, of personal experiences, and are also influenced by current trends in the literature. We have attempted, albeit not systematically, to collate the most commonly used genes in Fig 1. In Tables 4 and 5, we compared this “historical” list of genes associated with M1 or M2 polarization with the findings from our analysis, both in terms of RRA and for fold-change. As shown, a number of traditionally used genes do not appear robust as one would hope for when analysing the single stimuli.

**Table 4:** Comparison between the M1-“historical” murine genes with our analyses. *Numbers in parenthesis highlight the rank from our analysis*.

*GENE*	IFNγ		LPS		IFNγ+LPS	
	*RRA*	*avgFC*	*RRA*	*avgFC*	*RRA*	*avgFC*
***Tnf***	1.6E-03 (603)	3.2±1.0 (35)	9.7E-18 (12)	2.8±0.1 (2933)	4.9E-15 (8)	5.6±0.8 (77)
***Il1b***	n.s.	2.2±1.5 (529)	n.s.	3.5±0.2 (1681)	6.7E-07 (317)	7.8±0.8 (31)
***Il1a***	n.s.	0.9±1.5 (2778)	2.5E-14 (51)	5.0±0.1 (157)	2.0E-09 (121)	7.4±0.7 (29)
***Il12b***	1.6E-03 (607)	1.5 2.4 (1299)	1.5E-04 (773)	4.2±0.2 (575)	1.2E-05 (467)	9.4±0.9 (7)
***Il23a***	9.8E-08 (52)	0.5±0.5 (5432)	n.s.	4.7±0.1 (259)	n.s.	3.2± (254)
***Il6***	n.s.	2.4±1.2 (437)	2.5E-09 (251)	4.5±0.1 (361)	4.3E-06 (420)	8.8±1.1 (11)
***Ccl8***	2.4E-06 (131)	6.0±1.4 (42)	n.s.	3.6±0.8 (1215)	n.s.	0.6± (1254)
***Ccl7***	3.1E-04 (410)	2.1±0.9 (581)	2.8E-12 (107)	3.8±0.1 (1108)	6.9E-07 (323)	4.5± 0.6(119)
***Cxcl9***	6.2E-07 (93)	10.7±0.6 (3)	7.7E-12 (126)	4.0±0.1 (793)	1.7E-11 (51)	11.2±1.4 (2)
***Cxcl10***	1.0E-09 (18)	6.9±0.8 (29)	5.43E-16 (28)	4.5±0.1 (416)	1.4E-09 (117)	8.5±1.2 (16)
***Cxcl11***	1.2E-04 (322)	7.3±0.4 (25)	n.s.	1.6±0.1 (8187)	4.7E-07 (297)	10.3±1.0 (4)
***Nos2***	2.0E-07 (62)	9.9±1.1(6)	2.1E-12 (100)	3.5±0.1 (1389)	5.3E-11 (68)	11.2±1.5 (1)
***Socs3***	1.1E-05 (200)	4.7±0.8 (88)	4.1E-24 (1)	5.1±0.1 (153)	2.8E-14 (13)	6.9±0.9 (37)
***Cd86***	2.2E-04 (378)	3.0±0.6 (274)	n.s.	1.76±0.5(7432)	1.8E-09 (119)	3.7±0.7 (171)
***Cd80***	n.s.	-0.5±0.2 (23591)	6.4E-03 (1027)	2.0±0.3 (6282)	1.4E-02 (1162)	1.3±0.3 (837)
***Cd68***	n.s.	-0.2 ±0.2 (18377)	n.s.	-1.5±0.3 (28971)	n.s.	-0.2± (21589)
***Tlr4***	n.s.	0.3±0.3 (7821)	n.s.	0.3±0.5 (18843)	n.s.	-1.4± (27899)

**Table 5:** Comparison between the M2-“historical” genes with our analyses. *Numbers in parenthesis highlight the rank from our analysis*.

*GENE*	IL-4	
	*RRA*	*avgFC*
***Il10***	n.s.	-1.3±0.4 (32305)
***Tgfb1***	n.s.	-0.6±0.1 (28249)
***Il1rn***	n.s.	-0.2±0.2 (21470)
***Ccl16***	n.s.	NA
***Ccl22***	9.0E-12 (120)	4.1±0.5 (35)
***Ccl24***	2.8E-15 (60)	5.0±0.6 (21)
***Fizz (Retnla)***	5.27E-18 (36)	12.0 ± 1.0 (1)
***Arg1***	8.8E-28 (3)	8.5± 0.8 (3)
***Cd206 (Mrc1)***	2.69E-11 (126)	1.7±0.3 (151)
***Ym1 (Chil3)***	1.5E-25 (6)	6.2±0.8 (14)
***Ym2 (Chil4)***	1.21E-09 (179)	7.9±0.7 (6)

To overcome this arbitrariness, already known in the literature, in 2015 Jablonski et al. [17] used a single microarray dataset to propose a novel list of markers to delineate M1- and M2-signatures. This list was collated by choosing genes that were only up-regulated by M1- or M2-polarizing conditions and genes were ranked mainly on fold-change. As an M1 polarizing-agent, the Authors chose IFNγ+LPS while for M2, the Authors chose IL-4. While there are similarities between the two reports, there are also some clear differences. First, the work by Jablonski was performed using an Affymetrix microarray platform while we used RNAseq experiments. Second, our work expanded the realm of polarization, adding LPS and including also IFNγ alone. Most importantly, though, our work capitalized on multiple datasets to reduce noise and inter-experimental differences and used robust rank aggregation (RRA) as well as fold-change. The two signatures proposed, though, can be compared on the top ranked genes of IFNγ+LPS and IL-4 treatments. Of the top 20 highest fold-change genes reported in the present manuscript for IFNγ+LPS, 4 genes are in common with the top 17 highest fold-change genes from Jablonski. Of the top 20 highest fold-change genes reported in the present manuscript for IL-4, 5 genes are in common with the top 19 highest fold-change genes from Jablonski. Such concordance is high, in our view, also in consideration of the arbitrary cut-off of highest fold-change chosen (*i*.*e*. a gene not present in the top 20 might well be significantly regulated and be ranked below) and of the use of different technological platforms. Indeed, of the published list of 17 genes for IFNγ+LPS, only 5 genes, independently of ranking, did not result statistically significant in our RRA analysis (*Cxcl3*, *Ptges*, *Cd200*, *Ascl1*, *Ppap2a*). Of the published list for IL-4, only 6 did not result statistically significant in our RRA analysis (*Ear1*, *Ch25h*, *Chi3l3*, *Flt1*, *Chi3l4*, *Aqp9*).

A different way to use our analysis is to look at the most robust genes and understand their role and whether they have been object of previous investigations. Focusing solely on the top 20 lists, genes can be categorized in different categories: those for which a solid and consolidated role in macrophage polarization has been previously established (e.g. *Gbp2*, *Fgl2* [26], *Nampt* [27]), those that have so far not been object of investigation, and given the robustness of the change or the fold-change would deserve so (*Clic5*, *Styk1*, *Cbib*) and those which appear somehow contradictory to common knowledge (for example, *Cxcl16* is reported to be a gene that is upregulated both in M1 and M2, but our analysis depicts it as a M1-specific gene). In other words, our work represents a good starting point to fill the gaps of knowledge in macrophage polarization by highlighting genes not yet looked at or genes that require further understanding. Our work also provides a list of 28 genes that are highly significant (p<10^−7^) in all the three M1 stimuli and could be used as a starting point to define an agreed global M1 signature, when validated with other M1-polarizing agents (*e*.*g*. *Tnf*, *Cxcl10)*.

The present work should be read in light of the following weaknesses. First, we solely looked at murine macrophages and did not investigate whether the same changes are robust in the human counterparts. It is possible and likely that there will be differences between the two species due to a number of reasons, including species-specific genetic variations and transcriptional mechanisms. While a direct comparison between our analyses and the published human transcriptomic analyses (for example [28]) is possible, it would be best to compare lists generated via similar meta-analytical approaches to reduce noise and experimental variations. Second, we opted to analyse gene changes in a restricted time-frame, which is represented by the classical time-points used in experimental settings (between 2–8 hours for M1 polarizing agents and 18–24 hours for M2 agents) in macrophages from healthy mice using the most traditional M1- and M2-stimuli. Yet, (i) it is recognized that gene changes are dynamic; (ii) a number of other polarizing stimuli (for example, *IL-10*, *IL-13 TGF-b*, *PGE-2)* have been reported; and *(iii)* metabolic perturbations ((*e*.*g*. oxygen levels, pH, nutrient scarcity) within the microenvironment in health and disease (*e*.*g*. oxygen levels, pH, nutrient scarcity) profoundly affect the skewing and functional state of macrophages. Therefore, our data is a starting point but should be refined looking at those genes of interest from several different perspectives. Third, we solely looked at transcription, and did not investigate whether these changes translate in modifications in protein levels nor at the functional consequences of these changes. Last, this was an unbiased analysis that gave equal weight to the datasets retrieved, over which we had no control for quality, although given the systematic approach that made use of 39 experiments, noise and interexperimental variability were minimized.

Overall, the present paper used a meta-analytical approach to provide the genes that are most robustly changed upon macrophage polarization and the genes which appear to be the most up-regulated using fold-change. It is interesting to note that the two outcomes are not superimposable, and that very few genes are present in both lists. This would lead to consider RRA, rather than fold-change, as a better index for consistency across laboratories. The provided lists may be used as reference when investigating murine RNA-Seq experiments performed with other agents as well as a starting point to understand the role of those genes that have emerged and for which little is known at present.

## Supporting information

S1 File RRA score analysis of the up- and downregulated genes. (XLSX)

S2 File RRA score and avgFC of the up- and downregulated genes. (XLSX)

S3 File UPSTREAM regulators analysis. (XLSX)

S4 File IPA analysis. (XLSX)

S5 File Supplementary figures. (DOCX)

## References

[1] Sica A, Erreni M, Allavena P, Porta C. Macrophage polarization in pathology. Cell Mol Life Sci. 2015;72: 4111–4126. doi: 10.1007/s00018-015-1995-y 26210152 PMC11113543

[2] Ginhoux F, Guilliams M, Naik SH. Editorial: Dendritic Cell and Macrophage Nomenclature and Classification. Front Immunol. 2016;7: 168. doi: 10.3389/fimmu.2016.00168 27199991 PMC4852170

[3] Perdiguero EG, Geissmann F. The development and maintenance of resident macrophages. Nat Immunol. 2016;17: 2–8. doi: 10.1038/ni.3341 26681456 PMC4950995

[4] Murray PJ, Allen JE, Biswas SK, Fisher EA, Gilroy DW, Goerdt S, et al. Macrophage activation and polarization: nomenclature and experimental guidelines. Immunity. 2014;41: 14–20. doi: 10.1016/j.immuni.2014.06.008 25035950 PMC4123412

[5] Martinez FO, Gordon S. The M1 and M2 paradigm of macrophage activation: time for reassessment. F1000Prime Rep. 2014;6: 13. doi: 10.12703/P6-13 24669294 PMC3944738

[6] Biswas SK, Chittezhath M, Shalova IN, Lim J-Y. Macrophage polarization and plasticity in health and disease. Immunol Res. 2012;53: 11–24. doi: 10.1007/s12026-012-8291-9 22418728

[7] Porta C, Riboldi E, Ippolito A, Sica A. Molecular and epigenetic basis of macrophage polarized activation. Semin Immunol. 2015;27: 237–248. doi: 10.1016/j.smim.2015.10.003 26561250

[8] Travelli C, Colombo G, Mola S, Genazzani AA, Porta C. NAMPT: A pleiotropic modulator of monocytes and macrophages. Pharmacol Res. 2018;135: 25–36. doi: 10.1016/j.phrs.2018.06.022 30031171

[9] Sica A, Schioppa T, Mantovani A, Allavena P. Tumour-associated macrophages are a distinct M2 polarised population promoting tumour progression: potential targets of anti-cancer therapy. Eur J Cancer. 2006;42: 717–727. doi: 10.1016/j.ejca.2006.01.003 16520032

[10] Zhao C, Medeiros TX, Sové RJ, Annex BH, Popel AS. A data-driven computational model enables integrative and mechanistic characterization of dynamic macrophage polarization. iScience. 2021;24: 102112. doi: 10.1016/j.isci.2021.102112 33659877 PMC7895754

[11] Mantovani A, Marchesi F, Malesci A, Laghi L, Allavena P. Tumour-associated macrophages as treatment targets in oncology. Nat Rev Clin Oncol. 2017;14: 399–416. doi: 10.1038/nrclinonc.2016.217 28117416 PMC5480600

[12] Liu X, Zhang J, Zeigler AC, Nelson AR, Lindsey ML, Saucerman JJ. Network Analysis Reveals a Distinct Axis of Macrophage Activation in Response to Conflicting Inflammatory Cues. J Immunol. 2021;206: 883–891. doi: 10.4049/jimmunol.1901444 33408259 PMC7854506

[13] Chambers SEJ O’Neill CL, O’Doherty TM, Medina RJ, Stitt AW. The role of immune-related myeloid cells in angiogenesis. Immunobiology. 2013;218: 1370–1375. doi: 10.1016/j.imbio.2013.06.010 23932437

[14] Solinas G, Germano G, Mantovani A, Allavena P. Tumor-associated macrophages (TAM) as major players of the cancer-related inflammation. J Leukoc Biol. 2009;86: 1065–1073. doi: 10.1189/jlb.0609385 19741157

[15] Stark R, Grzelak M, Hadfield J. RNA sequencing: the teenage years. Nat Rev Genet. 2019;20: 631–656. doi: 10.1038/s41576-019-0150-2 31341269

[16] Hong M, Tao S, Zhang L, Diao L-T, Huang X, Huang S, et al. RNA sequencing: new technologies and applications in cancer research. J Hematol Oncol. 2020;13: 166. doi: 10.1186/s13045-020-01005-x 33276803 PMC7716291

[17] Jablonski KA, Amici SA, Webb LM, Ruiz-Rosado J de D, Popovich PG, Partida-Sanchez S, et al. Novel Markers to Delineate Murine M1 and M2 Macrophages. PLoS One. 2015;10: e0145342. doi: 10.1371/journal.pone.0145342 26699615 PMC4689374

[18] Kolde R, Laur S, Adler P, Vilo J. Robust rank aggregation for gene list integration and meta-analysis. Bioinformatics. 2012;28: 573–580. doi: 10.1093/bioinformatics/btr709 22247279 PMC3278763

[19] Ewels PA, Peltzer A, Fillinger S, Patel H, Alneberg J, Wilm A, et al. The nf-core framework for community-curated bioinformatics pipelines. Nat Biotechnol. 2020;38: 276–278. doi: 10.1038/s41587-020-0439-x 32055031

[20] Love MI, Huber W, Anders S. Moderated estimation of fold change and dispersion for RNA-seq data with DESeq2. Genome Biol. 2014;15: 550. doi: 10.1186/s13059-014-0550-8 25516281 PMC4302049

[21] Sheppe AEF, Kummari E, Walker A, Richards A, Hui WW, Lee JH, et al. PGE2 Augments Inflammasome Activation and M1 Polarization in Macrophages Infected With Salmonella Typhimurium and Yersinia enterocolitica. Front Microbiol. 2018;9: 2447. doi: 10.3389/fmicb.2018.02447 30429830 PMC6220063

[22] Wang W, Liang M, Wang L, Bei W, Rong X, Xu J, et al. Role of prostaglandin E2 in macrophage polarization: Insights into atherosclerosis. Biochem Pharmacol. 2023;207: 115357. doi: 10.1016/j.bcp.2022.115357 36455672

[23] Zhang F, Wang H, Wang X, Jiang G, Liu H, Zhang G, et al. TGF-β induces M2-like macrophage polarization via SNAIL-mediated suppression of a pro-inflammatory phenotype. Oncotarget. 2016;7: 52294–52306. doi: 10.18632/oncotarget.10561 27418133 PMC5239552

[24] Chuang Y, Knickel BK, Leonard JN. Regulation of the IL-10-driven macrophage phenotype under incoherent stimuli. Innate Immun. 2016;22: 647–657. doi: 10.1177/1753425916668243 27670945 PMC5292318

[25] Lopes RL, Borges TJ, Zanin RF, Bonorino C. IL-10 is required for polarization of macrophages to M2-like phenotype by mycobacterial DnaK (heat shock protein 70). Cytokine. 2016;85: 123–129. doi: 10.1016/j.cyto.2016.06.018 27337694

[26] Yan J, Zhao Q, Wang J, Tian X, Wang J, Xia X, et al. FGL2-wired macrophages secrete CXCL7 to regulate the stem-like functionality of glioma cells. Cancer Lett. 2021;506: 83–94. doi: 10.1016/j.canlet.2021.02.021 33676940 PMC8009861

[27] Colombo G, Travelli C, Porta C, Genazzani AA. Extracellular nicotinamide phosphoribosyltransferase boosts IFNγ-induced macrophage polarization independently of TLR4. iScience. 2022;25: 104147. doi: 10.1016/j.isci.2022.104147 35402885 PMC8990213

[28] Derlindati E, Dei Cas A, Montanini B, Spigoni V, Curella V, Aldigeri R, et al. Transcriptomic analysis of human polarized macrophages: more than one role of alternative activation? PLoS One. 2015;10: e0119751. doi: 10.1371/journal.pone.0119751 25799240 PMC4370704

[29] Bardou P, Mariette J, Escudié F, Djemiel C, Klopp C. jvenn: an interactive Venn diagram viewer. BMC Bioinformatics. 2014;15: 293. doi: 10.1186/1471-2105-15-293 25176396 PMC4261873

