# Supplementary material for: Getting everyone to agree on gene signatures for murine macrophage polarization in vitro
Source: PLoS One. 2024 Feb 8;19(2):e0297872. doi: 10.1371/journal.pone.0297872 (PMC10852255; doi:10.1371/journal.pone.0297872)
Supplement: S5 File — (DOCX) [file pone.0297872.s005.docx]

**SUPPLEMENTARY FIGURES**


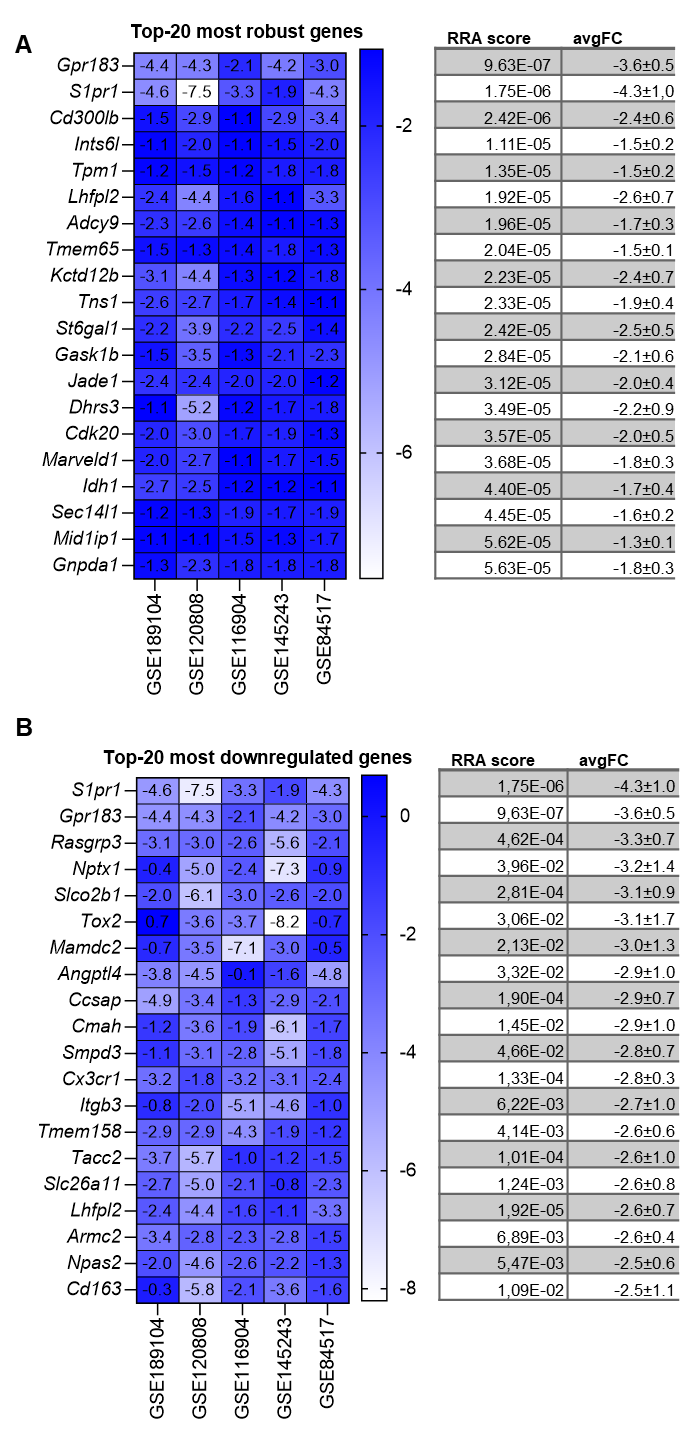


**Supplementary Figure 1. IFNγ down-regulated DEGs identified by RRA and fold-change analysis** (**A**) On the left, the heatmap of the five datasets showing the top 20 most robust genes down-regulated is depicted. Value in the boxes represents fold-change and shade of blue represents RRA score. On the right, the corresponding average RRA score and fold-change. (**B**) On the left, the heatmap of the five datasets showing the top 20 most down-regulated genes which also present a significant RRA score. Value in the boxes represents fold-change and shade of blue represents RRA score. On the right, the corresponding average RRA score and fold-change.


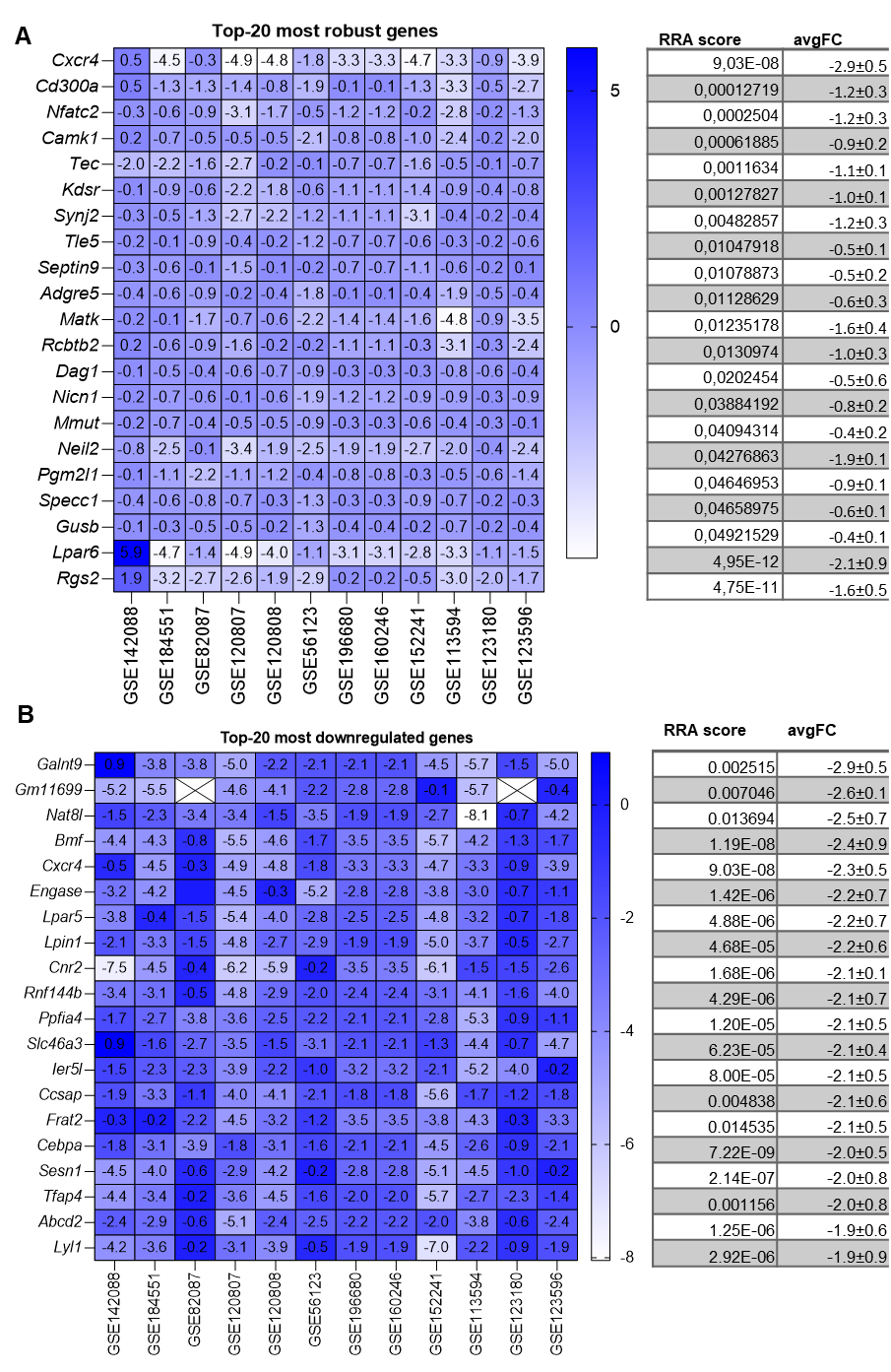


**Supplementary Figure 2. LPS down-regulated DEGs identified by RRA and fold-change analysis** (**A**) On the left, the heatmap of the twelve datasets showing the top 20 most robust genes down-regulated is depicted. Value in the boxes represents fold-change and shade of blue represents RRA score. On the right, the corresponding average RRA score and fold-change. (**B**) On the left, the heatmap of the twelve datasets showing the top 20 most down-regulated genes which also present a significant RRA score. Value in the boxes represents fold-change and shade of blue represents RRA score. On the right, the corresponding average RRA score and fold-change.


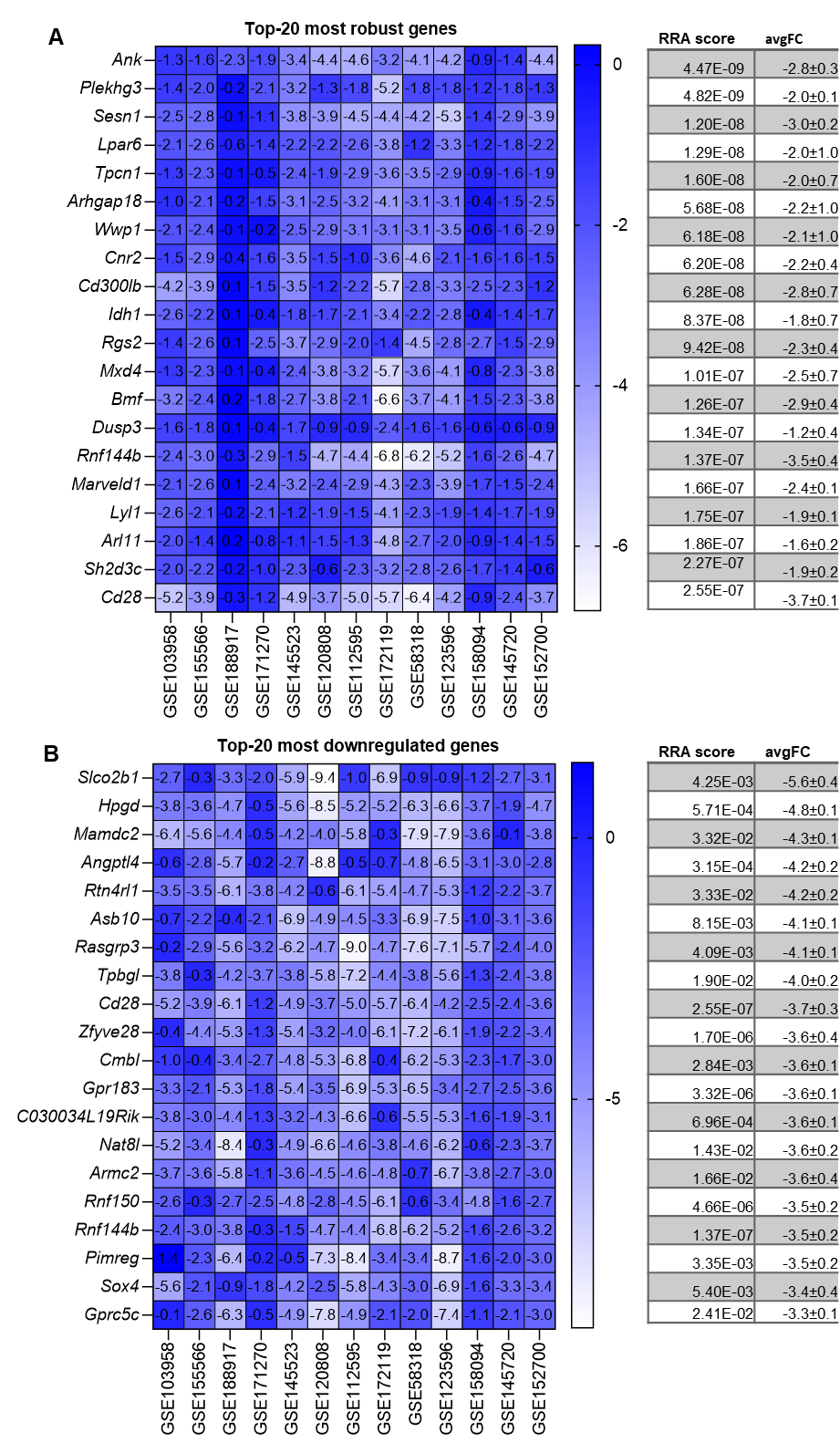


**Supplementary Figure 3. IFNγ+LPS down-regulated DEGs identified by RRA and fold-change analysis** (**A**) On the left, the heatmap of the thirteen datasets showing the top 20 most robust genes down-regulated is depicted. Value in the boxes represents fold-change and shade of blue represents RRA score. On the right, the corresponding average RRA score and fold-change. (**B**) On the left, the heatmap of the thirteen datasets showing the top 20 most down-regulated genes which also present a significant RRA score. Value in the boxes represents fold-change and shade of blue represents RRA score. On the right, the corresponding average RRA score and fold-change.


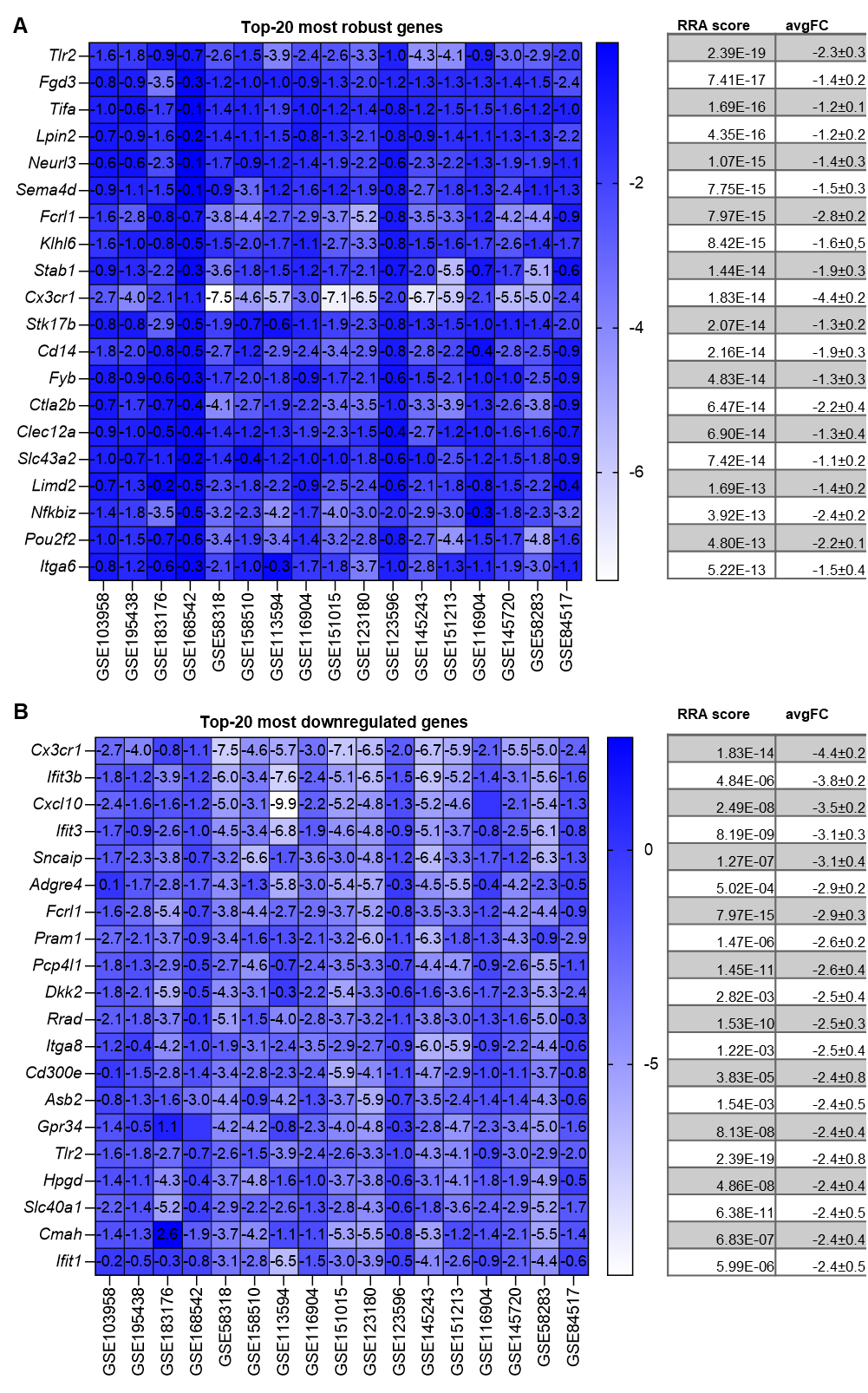


**Supplementary Figure 4. IL-4 down-regulated DEGs identified by RRA and fold-change analysis** (**A**) On the left, the heatmap of the seventeen datasets showing the top 20 most robust genes down-regulated is depicted. Value in the boxes represents fold-change and shade of blue represents RRA score. On the right, the corresponding average RRA score and fold-change. (**B**) On the left, the heatmap of the seventeen datasets showing the top 20 most down-regulated genes which also present a significant RRA score. Value in the boxes represents fold-change and shade of blue represents RRA score. On the right, the corresponding average RRA score and fold-change.
